# Supplementary material for: Autoimmune Limbic Encephalitis in Patients with Hematologic Malignancies after Haploidentical Hematopoietic Stem Cell Transplantation with Post-Transplant Cyclophosphamide
Source: Cells. 2023 Aug 11;12(16):2049. doi: 10.3390/cells12162049 (PMC10453524; doi:10.3390/cells12162049)
Supplement: Supplementary file 1 [file cells-12-02049-s001.zip › cells-2529799-supplementary.pdf]

Figure S1.

A

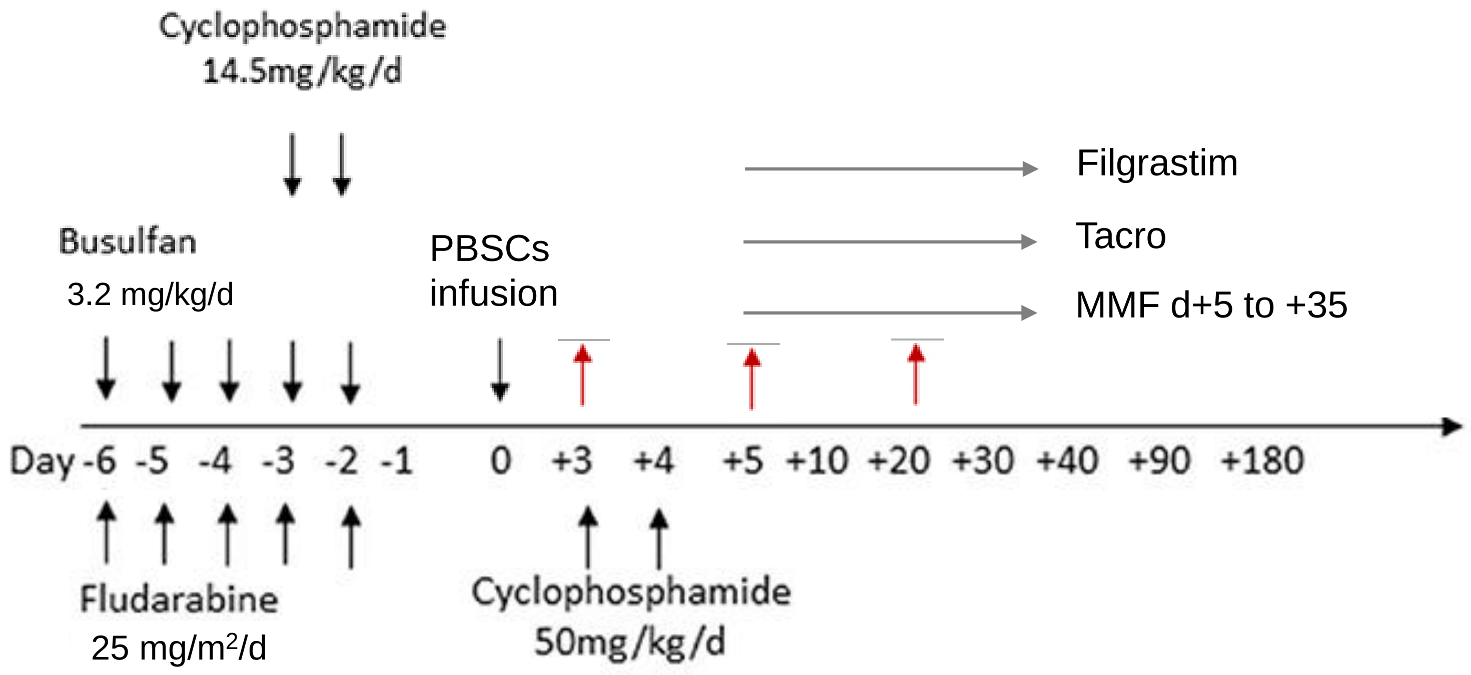

Myeloablating conditioning (MAC) regimen

B

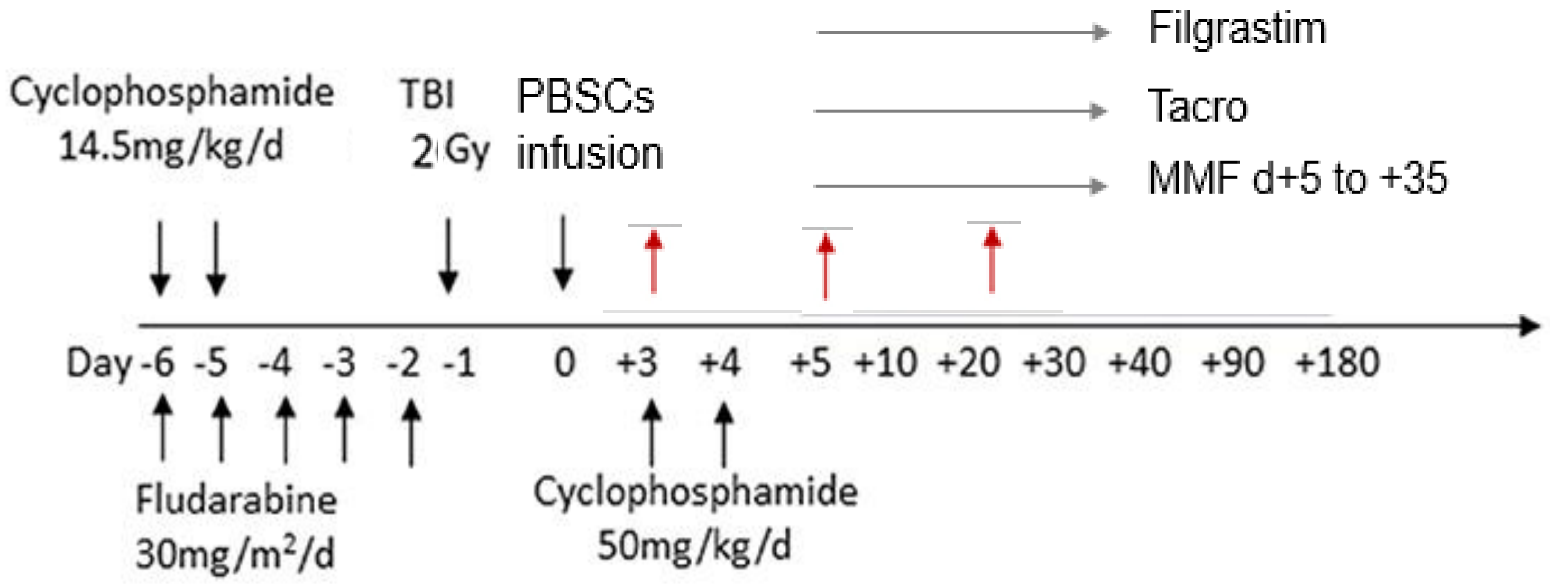

Reduced intensity conditioning regimen

Figure S1. Study designs for haploidentical HSCT with PTCy.
With PBSC infusion on day 0 and PTCy on post-transplant days 3 and 4, patients received either the (A) myeloablating conditioning regimen (N = 19) or (B) reduced intensity conditioning regimen in the week prior to transplant.

Figure S2.

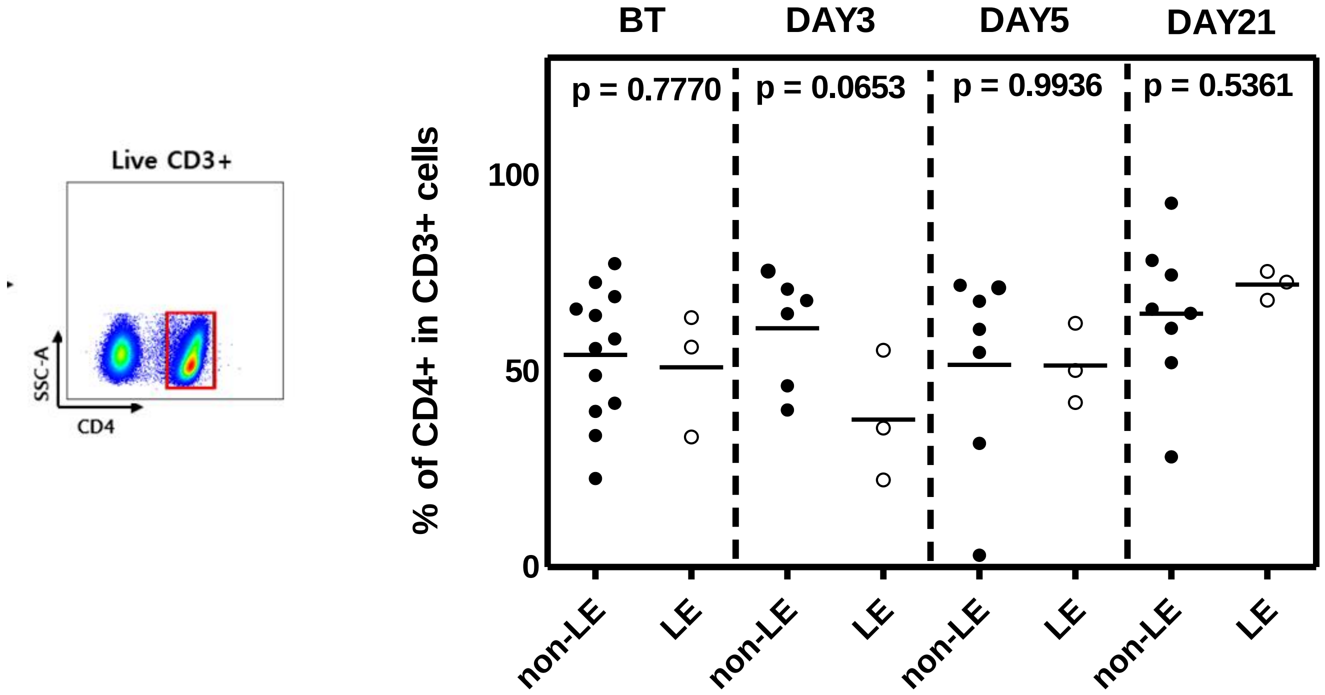

Figure S2. Frequency of CD4+ T cells in LE vs non-LE patients.
(A) Flow cytometry gating strategy. (B) Statistical analysis before (BT) and on days 3, 5, and 21 post-HCST**.** Statistical difference by two tailed t-test.

Figure S3.

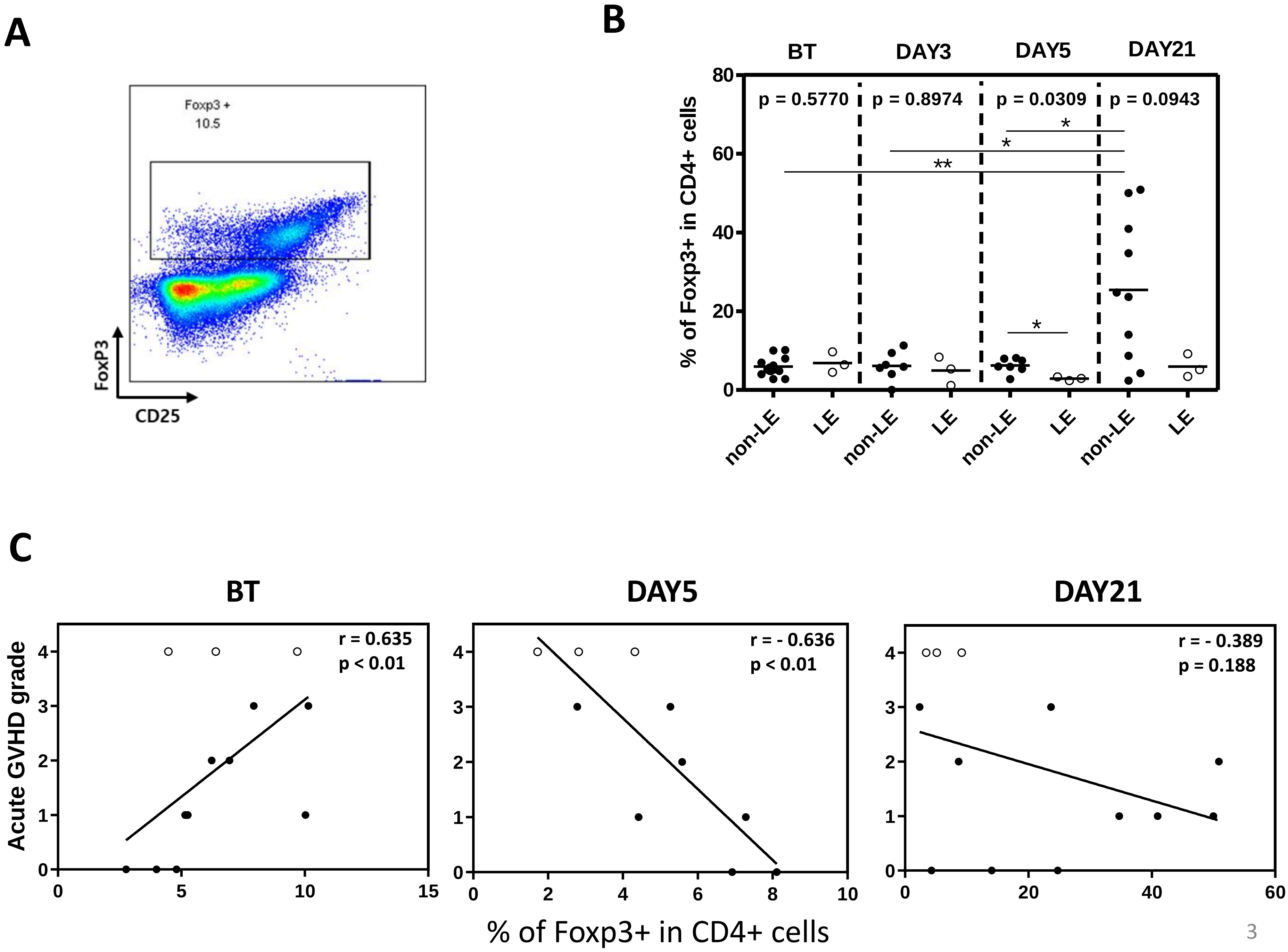

Figure S3. Frequency of CD4+Foxp3+ T cells in LE vs non-LE patients.
(A) Flow cytometry gating strategy. (B) Statistical analysis before (BT) and on days 3, 5, and 21 post-HCST. Statistical difference by unpaired two tailed t-test. *$p < 0.05$, **$p < 0.01$, ***$p < 0.001$. (C) Spearman correlation analysis with severity of acute GVHD at BT (left), post-HCST day 5 (mid), and post-HCST day 21 (right). The Spearman's correlation coefficient (r) and p value were indicated.

Figure S4.

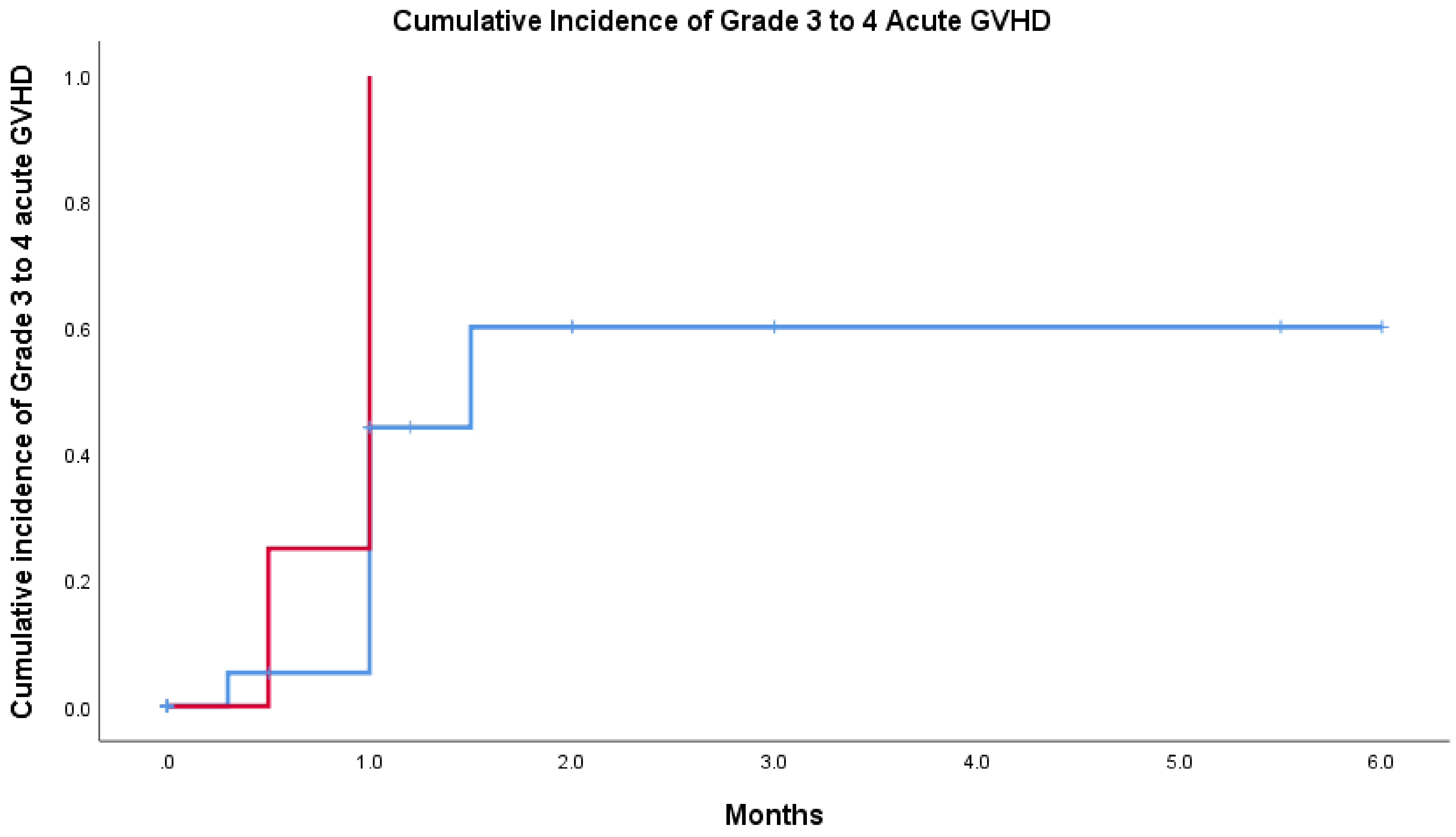

Figure S4. Cumulative incidence of grade 3 to 4 acute GVHD in immune-related LE and non-LE patients.

Comparison of cumulative incidence of severe (grade 3 to 4) acute GVHD between patients with (red) and without (blue) immune-related limbic encephalitis. Statistical difference by log-rank test (N=35, p=0.029)

Table S1.

Supplementary Table S1. Comparison of vasodilation and dyspnea between ALE and non-ALE patients

Limbic encephalitis	Yes (N=4)	No (N=31)	P value[§]
Vasodilation	3 (75%)	3 (9.7%)	0.001
Dyspnea	3 (75%)	8 (25.8%)	0.046

§ χ^2 test

Table S2.

Supplementary Table S2. Comparison of HLA types between ALE and non-ALE patients

Limbic encephalitis	Yes (N=4)	No (N=31)	P value[§]
A*0201 mismatched	2 (50%)	11 (35.5%)	0.572
B*4002 mismatched	2 (50%)	1 (3.2%)	0.002
DRB1*0802 mismatched	2 (50%)	1 (3.2%)	0.002

§ χ^2 test

Table S3.

Supplementary Table S3. Occurrence, organ manifestation and grading of acute GvHD for all patients (N=35)

Patient's Number	Occurrence of ac ute GVHD	Involved Organ of acute GVH D (Grade of individual organ)	Overall Grade of acute GVHD	Immune-related Limbic Encephalitis
1	No	No	0	No
2	Yes	Skin (4), Gut (4)	4	No
3	Yes	Skin (4)	3	No
4	Yes	Skin (3)	2	No
5	Yes	Gut (3)	3	No
6	Yes	Skin (3)	2	No
7	No	No	0	No
8	Yes	Skin (1)	1	No
9	Yes	Gut (3)	3	No
10	Yes	Skin (2), Liver (1)	2	No
11	Yes	Skin (2), Liver (3)	3	No
12	No	No	0	No
13	No	No	0	No
14	No	No	0	No
15	Yes	Skin (4)	3	No
16	Yes	Skin (4)	3	No
17	Yes	Skin (4)	3	No
18	No	No	0	No
19	No	No	0	No
20	Yes	Skin (4)	3	No
21	Yes	Skin (3), Liver (3)	3	Yes
22	No	No	0	No
23	No	No	0	No
24	Yes	Gut (4)	4	No
25	No	No	0	No
26	Yes	Skin (4), Liver (4)	4	Yes
27	No	No	0	No
28	Yes	Skin (2)	2	No
29	Yes	Skin (2)	2	No
30	No	No	0	No
31	Yes	Skin (4), Liver (4)	4	Yes
32	No	No	0	No
33	No	No	0	No
34	Yes	Skin (4), Liver (4)	4	Yes
35	Yes	Skin (2)	1	No
